# Evaluating the Urinary Exosome MicroRNA Profile in Prostate Cancer

**DOI:** 10.3390/genes17070802

**Published:** 2026-07-15

**Authors:** Beatriz Walter Rodriguez, Christopher J. Ricketts, Baris Turkbey, Peter A. Pinto, Maria J. Merino

**Affiliations:** 1 Laboratory of Pathology, Center for Cancer Research, National Cancer Institute, National Institutes of Health, Bethesda, MD 20892, USA; beatriz.walterrodriguez@nih.gov; 2 Urologic Oncology Branch, Center for Cancer Research, National Cancer Institute, National Institutes of Health, Bethesda, MD 20892, USA; chris.ricketts@nih.gov (C.J.R.); pintop@mail.nih.gov (P.A.P.); 3 Molecular Imaging Branch, Center for Cancer Research, National Cancer Institute, National Institutes of Health, Bethesda, MD 20892, USA; ismail.turkbey@nih.gov

**Keywords:** prostate cancer, urinary exosomes, miRNA, Gleason score, liquid biopsy, molecular markers

## Abstract

**Background/Objectives**: Prostate cancer is the second most frequent cancer among men and the 5th leading cause of cancer death among men worldwide. Identification of urine-derived biomarkers, such as exosomal miRNAs, in liquid biopsies for prostate cancer could be very beneficial for screening and active surveillance. **Methods**: Urine was collected from 42 patients with biopsy-proven evidence of prostate cancer and exosomes were extracted. Transcriptomic analysis was performed on the urine-derived exosomal miRNA and compared to the urine-derived exosomal miRNA profiles from 10 normal control donors and 15 von Hippel-Lindau (VHL) syndrome patients with clear cell renal cell carcinoma (ccRCC). **Results**: Urine-derived exosomal miRNA profiles of prostate patients were significantly different from normal control individuals. Significantly increased expression of miR-122-5p and decreased expression of miR-125-5p and miR-16-5p were observed in the urine-derived exosomes from prostate cancer patients. Significant upregulation of miR-30a-5p and downregulation of miR-320-5p, miR-320b, and miR-320c were observed in the urine-derived exosomes from both prostate cancer patients and VHL patients with ccRCC, indicating these miRNAs could be non-specific markers of urological cancer. Increased expression of miR-10a-5p and miR-30e-5p or miR-532-5p and miR-206 correlated with the presence of either extracapsular or perineural invasion, respectively. **Conclusions**: This study highlights the potential for urine-derived exosomal miRNA profiles to identify the presence of prostate cancer and predict clinical features, additionally showing that miRNA signals could be non-specific markers of urologic cancer types. Further validation studies are necessary to demonstrate the utility of urine-derived exosomal miRNA profiles as biomarkers for diagnosis or prognosis in prostate cancer.

## 1. Introduction

Prostate cancer (PC) is the second most frequent cancer among men and the 5th leading cause of cancer death among men worldwide [1]. The American Cancer Society estimated that in the United States 313,780 new cases will be diagnosed in 2025, with approximately 35,770 individuals dying from prostate cancer (https://cancerstatisticscenter.cancer.org/types/prostate (accessed on 4 August 2025)). Although the number of prostate cancer cases diagnosed had been declining, since 2014 the incidence rate has been increasing by ~3% per year overall [2]. Similarly, the mortality rate for prostate cancer is slowly reducing, but there remains a racial disparity with Black people having a two-fold higher mortality rate in comparison to White people [2]. Digital rectal examen (DRE) and the presence of prostate-specific antigen (PSA) are the tests currently used to detect prostate cancer in men 50 years and older, but increased screening opportunities would be beneficial for managing this disease. PSA is a highly sensitive test but has a low specificity for screening prostate cancer in symptomatic patients since it can also be detected in several benign prostatic conditions, such as prostatitis and benign prostatic hyperplasia. Therefore, the utility of using PSA alone to screen for prostate cancer is limited, and additional cancer-specific biomarkers are being investigated and recommended to improve cancer diagnosis with reduced misidentification of benign prostatic conditions [3,4].

During the development of more specific diagnostic tools, liquid biopsy has emerged as complementary to tissue biopsy for diagnosis, prognosis, and treatment in multiple cancers. This methodology assesses for the presence and composition of tumor cell-derived materials within bodily fluids, such as circulating tumor DNA or RNA (ctDNA, ctRNA), circulating tumor cells (CTCs), tumor-derived extracellular vesicles (EVs) including exosomes, and many other potential sample types that are currently emerging [5,6]. An additional advantage of liquid biopsy is that some bodily fluids, such as urine, can be obtained in a non-invasive manner that is beneficial to sample collection and can significantly increase the willingness of individuals to participate in regular testing [6]. Exosomes are a subpopulation of extracellular vesicles that are released in a greater abundance by cancer cells in comparison to normal cells. They carry proteins, lipids, DNAs, RNAs and microRNAs protected within a bilayer of lipids, and this cargo can participate in intercellular communication [7,8]. Studies of the specific composition of tumor-derived exosomes have been used to provide potential novel biomarkers in liquid biopsy. Urine liquid biopsies have been found to be useful to detect biomarkers for both urological and non-urological cancer [6,9,10]. In urological tumors, it has been suggested that tumor-derived genomic material excreted directly into the urinary tract has been suggested to increase specificity of detecting specific biomarkers.

In prostate cancer, tumor cells and cellular materials can be released into the prostatic fluids and then reach urine, enriching the urine for tumor-derived materials, such as exosomes containing both RNA and miRNA. Nilsson et al. demonstrated RNA from prostate cancer-derived exosomes in the urine of PC patients, finding cancer specific markers such as *PCA3* and *TMPRSS*-*ERG* [11]. The continuation of this idea led to the development of the ExoDx™ Prostate (IntelliScore) (EPI) test, an FDA-approved non-invasive urine test, that assesses the expression of three exosomal RNAs (*PCA3*, *SPDEF*, and *ERG*) that can be used in conjunction with PSA to help determine a man’s risk of having high-grade prostate cancer [12,13]. This highlights the potential for such tests and the need to identify additional biomarkers. The miRNA profile in urine of PC patients also shows distinct dysregulation, including overexpression of miR-21, miR-141-5p, miR-148a, and miR-375 in urinary exosomes [14,15]. Prediction models incorporating signatures based upon various dysregulated miRNAs have shown an increased statistical power to identify prostate cancer and to predict biochemical recurrence [16,17]. Further studies are needed to evaluate and expand upon the diagnostic and prognostic potential of the urinary exosome microRNA profiles.

In this study, we investigated the exosomal microRNA profiles by NGS in urine collected during follow-up from patients with varying degrees of prostate cancer and compared them with the microRNA profiles of urine-derived exosomes derived from healthy donor patients and from von Hippel-Lindau (VHL) syndrome patients with a burden of clear cell RCC, evaluated in a previous study [18]. This allowed us to look for potential biomarkers of prostate cancer and evaluate whether they were specific or could be reflective of urological cancer in general.

## 2. Materials and Methods

### 2.1. Patient Enrollment and Clinical Features

A total of 42 urines from prostate cancer patients, 10 urine samples from healthy donors, and 15 patients with VHL-associated ccRCC were evaluated and compared within this study. Patient recruitment, subsequent tissue procurement and use, and publication of data were approved by the Institutional Review Board of the National Cancer Institute on the NCI-16-C-0010, NCI-97-C-0147, or NCI-89-C-0086 protocols. All patients provided written, informed consent for the relevant protocols. Urine samples were collected at an outpatient clinic. The 10 urine samples from healthy donors and the 15 urine samples from presurgical VHL patients with VHL-associated ccRCC were presented in a previous publication, Walter-Rodriguez et al. 2024 [18]. For the prostate patients, pathological information was collected from the pathology reports, including Gleason score, Gleason Grade Group (GGG score), percentage of tumor involvement in the needle core biopsies and/or radical prostatectomy. Additionally, presence of tumor invasion at the margins, perineural, seminal vesicle and lymphovascular invasion, along with existence and percentage of prostate cancer cribriform growth pattern, amongst other variables. The clinical information, such as age, TNM stage, prostate cancer risk group (D’Amico classification system), presence of biochemical recurrence, was also included.

### 2.2. Urine Collection and Processing

Urines were processed within 4–6 h of collection. To remove the urinary cells and debris, the urine was centrifuged at 3000× *g* for 15 min (Allegra X30R centrifuge, Beckman Coulter, Indianapolis, IN, USA) and the cell-free urines (CFU) were separated in a new conical tube and stored at −80 °C temperature for analysis at a later stage.

### 2.3. Extracellular Fraction Isolation and Characterization

Using a 2 mL aliquot of CFU obtained previously, the Exo-Urine EV Isolation kit (System Biosciences, SBI, Palo Alto, CA, USA) was used according to the manufacturer instructions to isolate urinary-associated extracellular vesicles (EVs) by size exclusion chromatography. Validation of exosomes in the EV fraction had been previously performed as a component of our initial exosome study, and the results and methodology are described in Walter-Rodriguez et al. 2024 [18].

### 2.4. RNA Extraction

The RNA was extracted from the EV fraction using the SeraMir Exosome RNA Purification Column Kit (System Biosciences, SBI, Palo Alto, CA, USA). For quality control, gDNA contamination and RNA quantification for each sample were measured in a Bioanalyzer 2000 (Agilent Technologies, Santa Clara, CA, USA). Samples with more than 1 ng/5 uL were selected for library preparation for next-generation analysis (NGS).

### 2.5. miRNA Sequencing Analysis

42 prostate patient urine-derived RNA samples were pooled and sequenced on NextSeq 2000 P3 (Illumina Inc., San Diego, CA, USA) using QIAseq miRNA Library Prep (QIAGEN, Germantown, MD, USA) and single-end sequencing. All the samples had a percent of Q30 bases above 90% and have yields between 17 and 27 million pass filter reads. Samples were trimmed for adapters and low-quality bases using Trimmomatic before the alignment. Reads were trimmed to a maximum of 27 bases to carry out miRNA analysis using miRDeep2 with the hg38 reference [19]. miRDeep2 uses the Bowtie aligner for mapping, and the average mapping rate of all samples was 18%, reflecting a relatively low input concentration. This low mapping rate was acceptable but led to the detection of a limited number of miRNAs. This data was combined with the 10 urine samples from healthy donors and the 15 urine samples from presurgical VHL patients with VHL-associated ccRCC. Analysis was limited to the miRNAs identified within the prostate samples. The methods for urine collection, approximate times of storage duration and processing conditions were the same for both the prostate and the VHL samples, but they were sequenced in separate batches. Notably, the prostate samples all belong to males with an average age of 67.6 years old, while the VHL patients were a mixture of both male and female and were, on average, younger at the time of urine collection. The VHL samples were utilized to highlight non-specific enrichment of specific miRNAs in the urines of cancer-bearing individuals.

### 2.6. Bioinformatic and Statistical Analysis

All the bioinformatic and statistical analysis for NGS raw data was performed on the bulk RNAseq workflow available on the NIH Integrated Data Analysis Platform (NIDAP) for filtering and QC, PCA, expression heatmaps, differential expression analysis, and volcano plots. Differential expression analysis was predicated on the criteria of an adjusted *p*-value < 0.05 and |log2FC| > 1. Additional visualization and statistical analysis were performed using GraphPad Prism 10 (GraphPad Software, Boston, MA, USA) and the Mann–Whitney test (*p*-value < 0.05).

## 3. Results

### 3.1. Clinicopathological Features of Study Samples

A total of 42 patients with prostate cancer were included in our study. Patients had a median age of 67 years (range 56–77 years). All cases had a guided prostate biopsy, which was revised by a trained pathologist to confirm the diagnosis and evaluate the Gleason Score and Gleason Grade Group (GGG) (Table 1). Three of the 42 cases were GGG1 (GS3+3) (7.1%), 12/42 were GGG2 (GS3+4) (28.6%), 9/42 were GGG3 (GS4+3) (21.4%), 11/42 were GGG4: 1/42 was GS3+5 (2.4%) and 10/42 were GS4+4 (23.8%), finally 7/42 cases were GGG5 (GS4+5) (16.7%). In the needle biopsies, the percentage of tumor present within the cores and the presence of cribriform tumor growth pattern were assessed (Table 1). Additionally, a total of 18 cases (42.8%) underwent radical prostatectomy and pathological features, including the presence of extracapsular, perineural, lymphovascular, and seminal vesicle invasion along with the presence of cribriform growth pattern, were evaluated (Table S1). In one case all the pathological information was not available. Post surgery, four cases had a recurrence by imaging, five cases demonstrated biochemical recurrence, and one had an extra-prostatic recurrence. Of the 24 of 42 cases that had not undergone surgical treatment, 19 are currently under active treatment and no information is available for the remaining five cases. All urine samples required for exosome derivation were performed pre-surgery.

### 3.2. Evaluation of the Differences Between Urine-Derived Exosomal miRNA Profiles in Prostate Cancer Patients in Comparison to Normal Controls

**Figure 1:**
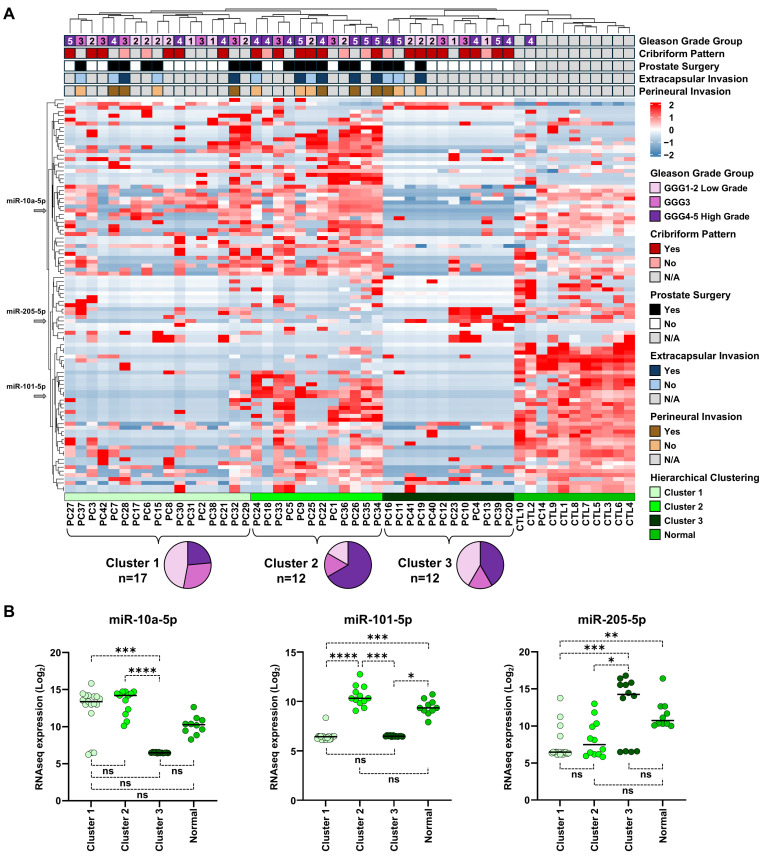
Heatmap of urine-derived exosomal miRNAs from prostate cancer patients. (**A**) Heatmap representation of the 100 most variable miRNAs across the entire cohort of 42 prostate cancer patients and 10 normal controls. Clinical features are presented above the heatmap. Three clusters of prostate cancer patients were defined by the dendrogram with pie-charts demonstrating the breakdown of the Gleason Grade Groups in each cluster. (**B**) Urine-derived exosomal miRNAs that demonstrated significant differential expression between clusters. ****—<0.0001, ***—<0.001, **—<0.01, *—<0.05, ns—not significant.

Principal component analysis (PCA) demonstrated that the urine-derived exosomal miRNA expression pattern from the prostate cancer patients was distinctly separate from the urine-derived exosomal miRNA expression pattern from the normal control individuals (Supplementary Figure S1A). While the prostate cancer patient urine-derived exosomal miRNA expression patterns were separated from normal, they demonstrated a considerable degree of variation and showed little evidence of this variation correlating with the Gleason Grade Group (GGG) (Figure S1A). Unsupervised clustering analysis of the 100 most variable miRNAs across the entire cohort created a heatmap with four distinct clusters, with the rightmost cluster representing all the urine exosomes derived from the normal control individuals and a single prostate cancer patient (PC14) (Figure 1A). Most prostate cancer patients were separated into three clusters, with cluster 1 (*n* = 17) and 3 (*n* = 12) demonstrating decreased exosomal miRNA expression in comparison to the normal urine, while cluster 2 (*n* = 12) showed generally increased levels of exosomal miRNA expression (Figure 1A). Specific miRNAs were observed to be differentially expressed within each cluster, including miR-10a-5p, miR-101-5p, and miR-205-5p (Figure 1B). Cluster 2 correlated with an increased fraction of higher GGG prostate tumors (GGG4-5) and a greater degree of surgery, likely correlated to the increased grade (Figure 1). This suggests that increased expression of selected exosomal miRNA could correlate with higher grade and, in patients that had surgery, with varying degrees of invasion. Clusters 1 and 3 suggest that decreased exosomal miRNA expression could correlate with the presence of prostate cancer.

### 3.3. Potential Diagnostic Markers Within Urine-Derived Exosomal miRNAs in Prostate Cancer Patients

**Figure 2:**
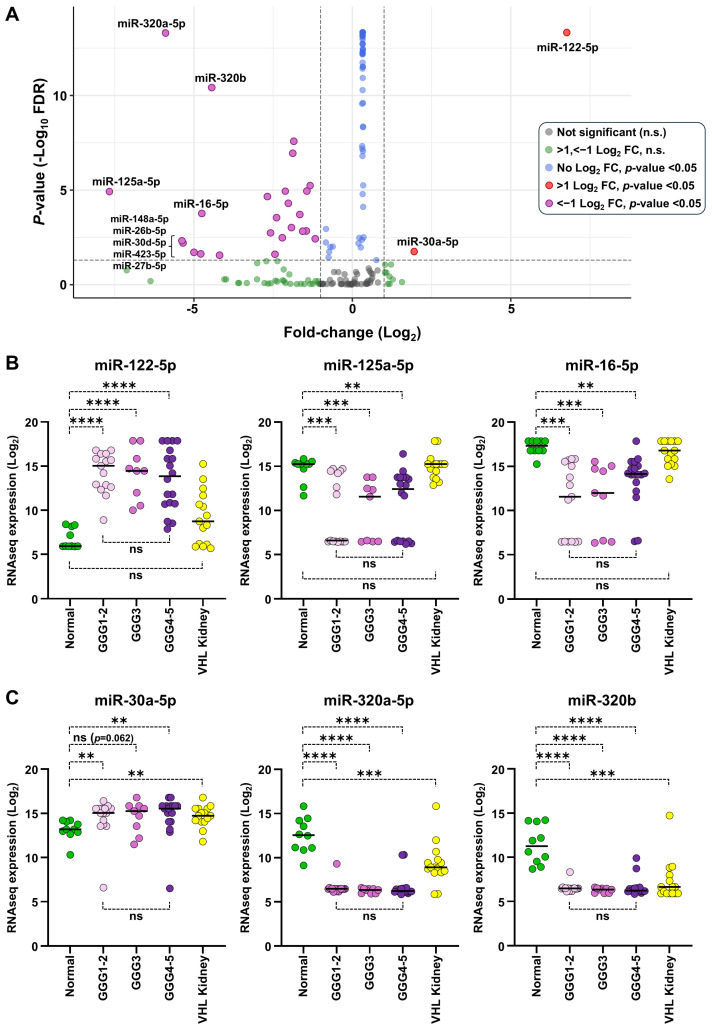
Differential miRNA expression analysis comparing urine-derived exosomal miRNAs from prostate cancer patients, normal controls, and VHL patients with ccRCC. (**A**) Volcano plot comparing urine-derived exosomal miRNAs from 42 prostate cancer patients with 10 normal controls. *p*-values were FDR adjusted and a value of <0.05 was considered significant. (**B**) Urine-derived exosomal miRNAs that demonstrated significant differential expression in prostate cancer patients in comparison to both normal controls and VHL syndrome patients with ccRCC. (**C**) Urine-derived exosomal miRNAs that demonstrated significant differential expression in both prostate cancer patients and VHL syndrome patients with ccRCC in comparison to normal controls. ****—<0.0001, ***—<0.001, **—<0.01, ns—not significant.

A comparison of the urine-derived exosomal miRNAs from the prostate cancer patients with the urine-derived exosomal miRNAs from the normal control individuals was performed and significant miRNAs were defined as having a two-fold difference in expression and an adjusted *p*-value of less than 0.05 (Log2 fold change > 1 or >−1, *p*-value < 0.05) (Figure 2A). This identified two miRNAs with increased expression in prostate cancer urines, miR-122-5p and miR-30a-5p, and 25 miRNAs with decreased expression in prostate cancer urines, including miR-320-5p, miR-320b, miR-125a-5p, and miR-16-5p (Figure 2A, Table S2). In a previous study, exosomal miRNAs had been extracted in an identical manner from 15 patients with VHL syndrome who had clear cell renal cell carcinoma [18]. To investigate the specificity of these differences, a similar comparison was performed between the urine-derived exosomal miRNAs from the VHL patients and urine-derived exosomal miRNAs from the same normal control individuals, highlighting 16 upregulated and six downregulated miRNAs (Figure S2A). Notably, miR-30a-5p was significantly upregulated in both sets of patients and miR-320-5p, miR-320b, and miR-320c were significantly downregulated across patients, while upregulation of miR-122-5p and downregulation of miR-125-5p and miR-16-5p was unique to the prostate cancer patients. This was confirmed using Mann–Whitney tests (*p*-value < 0.05) to account for the relatively small sample size (Figure 2B,C).

Due to the clustering shown in Figure 1, the urine-derived exosomal miRNAs from just the cluster 2 prostate cancer patients were compared to the urine-derived exosomal miRNAs from the normal control individuals, identifying 15 upregulated miRNAs and 36 downregulated miRNAs (Table S2). When these miRNAs were compared with the differentially expressed miRNAs in the VHL patients, all six downregulated miRNAs from the VHL patients were shared with cluster 2 of the prostate cancer patients, as well as six additional upregulated miRNAs (Figure S2B). This suggests that a considerable fraction of the differentially expressed miRNAs might be shared across urological cancers and not be cancer subtype specific. This highlights the potential importance of the differences in miR-122-5p and miR-125-5p that were tumor type-specific in this analysis but would require further confirmation in comparison to other cancer subtypes.

### 3.4. Potential Clinically Associated Markers Within Urine-Derived Exosomal miRNAs in Prostate Cancer Patients

**Figure 3:**
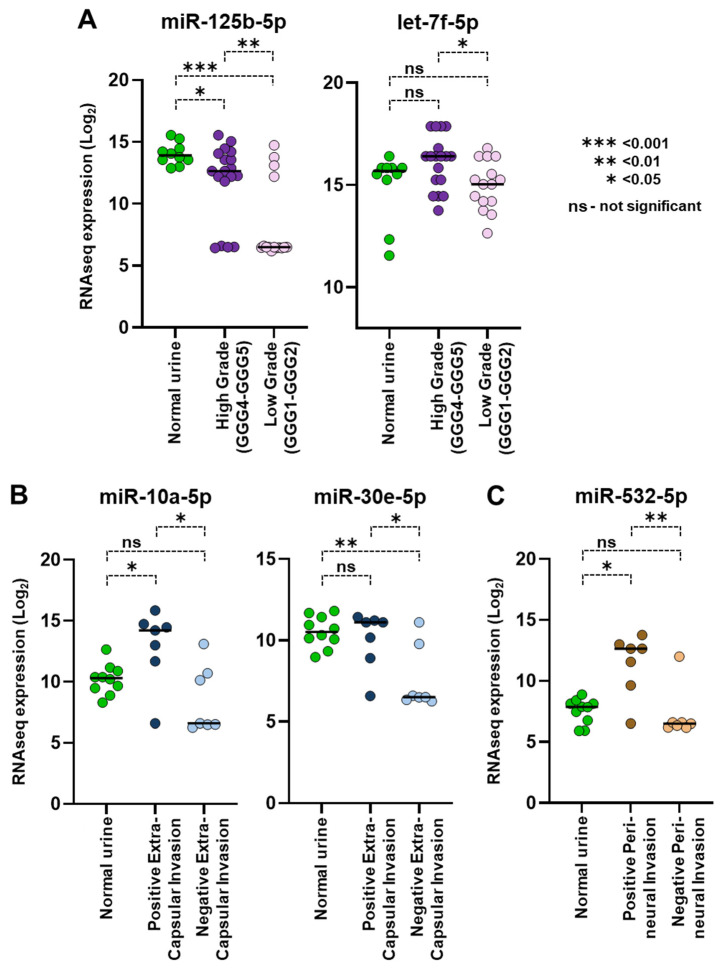
Differential expression analysis is associated with clinical features in prostate cancer patients. (**A**) Differentially expressed miRNAs associated with high grade tumors (GGG4/5). (**B**,**C**) Differentially expressed miRNAs associated with either extracapsular or perineural invasion. ***—<0.001, **—<0.01, *—<0.05, ns—not significant.

Biopsy analysis provided both the Gleason Grade Group (GGG) and presence of cribriform pattern for most cases. To evaluate potential markers of higher GGG disease, we compared the exosomal miRNA patterns from the 15 low GGG patients (3 GGG1 + 12 GGG2) with the 18 high GGG patients (11 GGG4 + 7 GGG5) (Table 1). Four exosomal miRNAs demonstrated significantly different levels of expression in the urine from prostate cancer patients with higher GGG in comparison to lower GGG cases, miR-125-5p, Let-7f-5p, miR-1269a, and miR-943-5p (Figure 3A and Figure S3A). Both miR-125-5p and Let-7f-5p demonstrated increased expression in the high GGG cases, but neither showed increased expression in comparison to normal urine and both the high and low GGG cases had significantly lower expression of miR-125-5p than normal urine. Conversely, both miR-1269a and miR-943-5p showed significantly increased expression in the low GGG cases in comparison to both high GGG cases and normal urine. While sufficient data was available for the presence of cribriform pattern, no significant differences in miRNA expression were observed between the 20 patients with evidence of cribriform pattern and the seven patients without (Table 1). Although some differences were observed using Welch’s *t*-test, these were not confirmed by Mann–Whitney tests and so were considered non-significant (Figure S3B).

Of the 42 prostate tumor cases, 18 patients underwent radical prostatectomy allowing for the evaluation of features such as margin positivity and localized invasion. In the cases of margin analysis and lymphovascular or seminal vesicle invasion, there were too few reported positive cases to allow for analysis of differential miRNA signals (Table S1). However, both extracapsular and perineural invasion were observed in seven cases and absent in another seven cases allowing for a limited evaluation, although there was considerable overlap within these cases.

Four exosomal miRNAs demonstrated significantly different levels of expression in the urine from prostate cancer patients with positive extracapsular invasion, miR-10a-5p, miR-30e-5p, miR-122-5p, and let-7e-5p, in comparison to negative cases (Figure 3B and Figure S3C). Three of these, miR-10a-5p, miR-30e-5p, and let-7e-5p, showed enrichment for increased expression in the positive extracapsular invasion cases, but only miRNA-10-5p showed increased expression in comparison to normal urine. Interestingly, miR-122-5p, which was also a general marker for prostate cancer, showed a significantly increased expression in the negative extracapsular invasion cases in comparison to both positive perineural invasion cases and normal urine.

Three exosomal miRNAs demonstrated significantly increased expression in the urine from prostate cancer patients with positive perineural invasion, miR-532-5p, miR-206, and miR-125b-5p (Figure 3C and Figure S3D). For both miR-532-5p and miR-206, positive perineural invasion cases were enriched for increased expression in comparison to both negative perineural invasion cases and normal urine. While miR-125b-5p was generally less expressed in negative perineural invasion cases when compared to both positive perineural invasion cases and normal urine. Notably, miR-125b-5p was also a marker of higher GGG and miR-206 was a marker for extracapsular invasion that nearly reached statistical significance, correlating with the overlap of these features within patient tumors (Figure S3C).

## 4. Discussion

Liquid biopsy has great potential to influence the diagnosis, surveillance and management of many cancers due to the less invasive nature of the technique, with urine analysis providing a particularly attractive method. Patients are more likely to opt for regular testing using such techniques and the cost of these tests is likely to reduce over time. These techniques are particularly relevant to prostate cancer wherein a large number of patients need to be screened for a very common cancer type, and many patients may be on active surveillance due to the low-grade nature of their disease but require regular evaluation for any advancement of their cancer. For instance, an important aspect of the clinical management of any prostate cancer is the Gleason Grade Group (GGG), which is currently defined by biopsy. Identifying markers of the presence of increased GGG in urine would be advantageous as these tests could be performed more regularly and influence when the next biopsy might be performed on patients undergoing active surveillance or whether surgical options are required.

The use of urine-based testing for prostate cancer has already proven an attractive option. The MyProstateScore 2.0 (MPS2) test is a urine-based test that evaluates the expression of 18 mRNAs, 13 prostate cancer-specific gene markers (*PCGEM1*, *SPON2*, *TRGV9*, *PCA3*, *OR51E2*, *CAMKK2*, *TFF3*, *PCAT14*, *TMSB15A*, *HOXC6*, *ERG*, *TMPRSS2*:*ERG*, and *KLK4*), four high-grade cancer-specific genes (*APOC1*, *B3GNT6*, *NKAIN1*, and *SCHLAP1*), and the reference gene *KLK3* [20]. This test can predict the presence of GGG2 or greater prostate cancer, while excluding GGG1 prostate cancer or benign disease, and provide evidence for the need for further standard biopsy-based evaluation. Highly relevant to this study are the miR Sentinel™ prostate cancer tests that interrogate the expression of small noncoding RNAs (sncRNA) isolated from urinary exosomes [21]. The Sentinel HG test is designed for individuals currently diagnosed with prostate cancer and evaluates the expression of 122 miRNAs and 25 small nucleolar (sno)RNAs to differentiate between lower (GG1-2) and higher-grade disease (GG3-5) disease. While the Sentinel CS test can be used to surveil patients over long periods of time and evaluates expressions of 130 miRNAs and 66 snoRNAs to classify patients with either low-grade (GG1) or intermediate- and high-grade cancer (GG2-5) to influence further clinical assessment. Thus, further improvements in this field could prove very beneficial.

Within this study, the urine-derived exosomal miRNA profiles from prostate cancer patients were distinctly different from those acquired from normal control individuals, but this does not necessarily mean these observations were specific to prostate cancer. We observed two significantly upregulated miRNAs, including miR-30a-5p, which were also upregulated in the VHL patients with ccRCC and has previously been shown as a biomarker for prostate cancer [22]. Within this study, miR-122-5p was uniquely upregulated in prostate cancer patients and has been previously shown to be upregulated in prostate cancer tumors [23,24]. While urine-derived exosomal expression levels of miR-122-5p have not yet been reported in prostate cancer, increased expression in the urine of sporadic ccRCC cases has been reported [25]. Similarly, miR-320-5p, miR-320b, and miR-320c were downregulated in both the prostate cancer and VHL ccRCC patients and these miRNAs have been previously reported to be lower in the serum and urine of patients with prostate cancer and the urine of the VHL ccRCC patients [18,26,27]. Two miRNAs, miR-125-5p and miR-16-5p, were only observed in the urine of the prostate patients and miR-125-5p has been previously shown to be downregulated in prostate cancer patient urines [28]. This data highlights the potential for diagnostic markers for prostate cancer within urine-derived exosomes but emphasizes the observation of shared biomarkers between urological cancers and that broader miRNA profiles may be more effective than single biomarkers, such as seen in the miR Sentinel™ prostate cancer tests.

Additionally, this study identified four miRNAs that were differentially expressed between higher and lower GGG prostate cancers and seven miRNAs that were differentially expressed dependent upon the presence of either extracapsular or perineural invasion, including miR-122-5p and miR-125-5p that were observed as generally increased or decreased, respectively, within prostate cancer urine exosomes. No published confirmatory data was found for the GGG-associated miRNA. Of the markers associated with extracapsular or perineural invasion, increased miR-10a-5p and miR-206 expression in prostate cancer has been associated with higher grade prostate tumor and miR-532-5p expression level in urine exosomes has been previously proposed as a predictive biomarker for biochemical recurrence (BCR) of intermediate-risk prostate cancer [23,24,29]. This data highlights the potential utility of these biomarkers, but the relatively small numbers of samples mean that confirmation of significant observations is required from larger cohorts.

The limitations of this study are the relatively small cohort, the use of a single extraction methodology, the relatively low input levels derived from the prostate samples, and the lack of a confirmatory cohort. Considering the data from this initial study, improvements to our further studies would be made by increasing patient numbers, enhancing miRNA extraction, increasing sequencing sample input, and potentially expanding analysis to evaluate all the RNA types derived from the exosomes. While the internal comparison of miRNA from urine-derived exosomes from prostate cancer patients and VHL patients with ccRCC does provide data from two urological cancer bearing cohorts, some important observations may be obscured due to the differences between the patient populations and batch effect. The prostate patients were all male, while the VHL patients represent both genders, and the prostate patients were on average older than the VHL patients at sample collection. Additionally, confirmation by comparison to other published studies is also limited as the experimental methodologies for evaluating urine-derived exosomal miRNA profiles can vary greatly between studies, such as using different urine fractions, exosome isolation methods and controls. Thus, these studies are useful to increase our knowledge and provide potential biomarkers, but require further validation within multiple, larger cohorts utilizing several methodologies to provide reliable conclusions.

This study confirms the potential for urine-derived exosomal miRNA profiling to aid in the diagnosis of prostate cancer and predict some of the clinical features, such as Gleason Grade Grouping and degree of invasion. With the appropriate confirmatory studies, this data could advance the refinement and expansion of existing liquid biopsy-based testing for the presence and grading of prostate cancer, such as the miR Sentinel™ prostate cancer tests, and improve the future management and surveillance of prostate cancer patients.

## Figures and Tables

**Table 1:** Demographic and pathological information.

Age (years)			
median 67 range 56–77			
Biopsy Gleason Grade Group	*n*	%	Total
GGG1 (GS3+3)	3	7.14	42
GGG2 (GS3+4)	12	28.57	
GGG3 (GS4+3)	9	21.43	
GGG4 (GS3+5)	1	2.38	
GGG4 (GS4+4)	10	23.81	
GGG5 (GS4+5)	7	16.67	
Percent of core with tumor (%)			
0	2	4.76	42
1 to 10	2	4.76	
11 to 24	6	14.29	
25 to 50	7	16.67	
51 to 74	9	21.43	
75 to 100	8	19.05	
n/a	8	19.05	
Presence of cribriform pattern			
yes	20	47.62	42
no	7	16.67	
n/a	15	35.71	
Percent of cribriform pattern in core with tumor (%)			
0	7	16.67	42
1 to 10	5	11.90	
11 to 24	6	14.29	
25 to 50	3	7.14	
51 to 74	4	9.52	
75 to 100	1	2.38	
n/a	16	38.10	

## Data Availability

The original contributions presented in the study are included in the article/Supplementary Materials; further inquiries can be directed to the corresponding author.

## Supplementary Materials

The following supporting information can be downloaded at: https://www.mdpi.com/article/10.3390/genes17070802/s1, Figure S1: Principal Component Analysis of urine-derived exosomal miRNAs from prostate cancer patients; Figure S2: Differential expression analysis of urine-derived exosomal miRNAs from VHL patients with ccRCC and prostate cancer patients; Figure S3: Additional differential expression analysis associated with clinical features in prostate cancer patients; Table S1: Pathological information; Table S2: Differential urine-derived exosomal miRNA analysis.

## Abbreviations

The following abbreviations are used in this manuscript:

ccRCC	clear cell renal cell carcinoma
PSA	prostate-specific antigen
CTC	circulating tumor cells
GGG	Gleason Grade Group
