# Supplementary material for: Evaluating the Urinary Exosome MicroRNA Profile in Prostate Cancer
Source: Genes (Basel). 2026 Jul 15;17(7):802. doi: 10.3390/genes17070802 (PMC13409727; doi:10.3390/genes17070802)
Supplement: Supplementary file 1 [file genes-17-00802-s001.zip › genes-4404199-supplementary.pdf]

Supplementary Data

Table S1: Pathological information.

Radical prostatectomy	*n*	%	total
yes	18	42.86	42
no	23	54.76	
n/a	1	2.38	
Extracapsular invasion			18
positive	7	38.89	
negative	7	38.89	
n/a	4	22.22	
Margin			18
positive	2	11.11	
negative	12	66.67	
n/a	4	22.22	
Perineural invasion			18
positive	7	38.89	
negative	7	38.89	
n/a	4	22.22	
Lymphovascular invasion			18
positive	2	11.11	
negative	12	66.67	
n/a	4	22.22	
Seminal vesicle invasion			
positive	2	11.11	18
negative	12	66.67	
n/a	4	22.22	
Presence of Cribriform Growth Pattern			18
yes	5	27.78	
no	4	22.22	
n/a	9	50.00	

Table S2: Differential urine-derived exosomal miRNA analysis.

Comparison	Gene	Tumor-Normal_FC	Tumor-Normal_logFC	Tumor-Normal_tstat	Tumor-Normal_pval	Tumor-Normal_adjpval
Prostate urine vs normal urine	hsamiR1225p	108.3669417	6.759780907	11.31448443	1.64E-15	4.55E-14
Prostate urine vs normal urine	hsamiR30a5p	3.872405487	1.953230028	2.718100154	0.008950922	0.017901844
VHL urine vs normal urine	hsamiR3355p	15.05015121	3.911706077	5.5555425	9.85E-06	4.68E-04
VHL urine vs normal urine	hsamiR1825p	13.94848906	3.802036949	3.385485195	0.002415491	0.028683955
VHL urine vs normal urine	hsamiR1835p	12.84431835	3.683058424	6.374779895	1.29E-06	1.23E-04
VHL urine vs normal urine	hsamiR2415p	10.41801541	3.38100857	3.062216602	0.005304935	0.045815344
VHL urine vs normal urine	hsamiR2045p	9.35286489	3.225408347	3.738919065	0.00100119	0.015852168
VHL urine vs normal urine	hsamiR196a5p	6.502221811	2.700932772	3.530427643	0.001686896	0.022584427
VHL urine vs normal urine	hsalet7e5p	5.349258828	2.419339011	4.017573059	4.95E-04	0.009402914
VHL urine vs normal urine	hsalet7g5p	5.132547629	2.35967511	3.876451723	7.08E-04	0.012224268
VHL urine vs normal urine	hsamiR29b15p	4.774227785	2.255267401	3.320532593	0.002833856	0.029912925
VHL urine vs normal urine	hsamiR29b25p	4.692751088	2.230433939	3.255792454	0.003320359	0.033203592
VHL urine vs normal urine	hsamiR10a5p	4.443413746	2.151668483	4.084538238	4.17E-04	0.009402914
VHL urine vs normal urine	hsamiR315p	3.663254928	1.873126102	3.508149086	0.001782981	0.022584427
VHL urine vs normal urine	hsamiR200a5p	3.509753989	1.81136991	4.028872034	4.81E-04	0.009402914
VHL urine vs normal urine	hsamiR1925p	3.110244337	1.637027921	3.601031669	0.001414614	0.020675127
VHL urine vs normal urine	hsamiR30a5p	3.042497713	1.605256178	4.117051372	3.84E-04	0.009402914
VHL urine vs normal urine	hsamiR10b5p	3.013282096	1.591335741	3.191084627	0.003886851	0.035166751
Cluster 2 urine vs normal urine	hsamiR664a5p	28.75185971	4.845583369	3.257947137	0.004754593	0.022007556
Cluster 2 urine vs normal urine	hsamiR3355p	22.5444019	4.494697331	2.905653129	0.01003535	0.02345107
Cluster 2 urine vs normal urine	hsamiR196a5p	20.2429493	4.339347594	3.305307464	0.004297078	0.022007556
Cluster 2 urine vs normal urine	hsamiR1835p	17.99996916	4.16992253	3.181602494	0.005595195	0.022007556
Cluster 2 urine vs normal urine	hsamiR6285pp	13.11426141	3.713064653	3.69869481	0.00184866	0.011708179
Cluster 2 urine vs normal urine	hsamiR4555p	12.1860526	3.607158967	3.365679065	0.003776478	0.021158549
Cluster 2 urine vs normal urine	hsamiR30655p	11.80530491	3.561363399	3.597588421	0.002296809	0.014077215
Cluster 2 urine vs normal urine	hsamiR200c5p	11.47908467	3.520935703	2.963693253	0.008880919	0.022802361
Cluster 2 urine vs normal urine	hsamiR148b5pp	9.679107454	3.274874017	3.751311604	0.001651211	0.010992124
Cluster 2 urine vs normal urine	hsamiR10a5p	8.806558733	3.138578379	3.743884796	0.001677745	0.010992124
Cluster 2 urine vs normal urine	hsamiR19b15p	7.771699875	2.958230188	3.887824237	0.001232003	0.009003102
Cluster 2 urine vs normal urine	hsamiR19b25p	7.771699875	2.958230188	3.887824237	0.001232003	0.009003102
Cluster 2 urine vs normal urine	hsamiR10b5p	5.630653057	2.493302259	3.1026668	0.006617857	0.022007556
Cluster 2 urine vs normal urine	hsamiR1925p	5.456098624	2.447869723	3.499130904	0.002837158	0.016845625
Cluster 2 urine vs normal urine	hsamiR30a5p	4.75635639	2.249856819	4.110443391	7.65E-04	0.006922252

Comparison	Gene	Tumor-Normal_FC	Tumor-Normal_logFC	Tumor-Normal_tstat	Tumor-Normal_pval	Tumor-Normal_adjpval
Prostate urine vs normal urine	hsamiR225p	-2.269050956	-1.182089008	-3.323641499	0.00165078	0.003647072
Prostate urine vs normal urine	hsamiR1455p	-2.524149848	-1.335797559	-5.363396199	2.00E-06	5.68E-06
Prostate urine vs normal urine	hsamiR1505p	-2.73137052	-1.449625034	-5.157915436	4.13E-06	1.14E-05
Prostate urine vs normal urine	hsamiR2215p	-2.763384491	-1.46643631	-3.652081114	6.14E-04	0.001457671
Prostate urine vs normal urine	hsalet7d5p	-2.978002767	-1.574345094	-3.642533478	6.32E-04	0.001482631
Prostate urine vs normal urine	hsamiR285p	-3.15240332	-1.656452126	-4.304213053	7.61E-05	1.90E-04
Prostate urine vs normal urine	hsamiR985p	-3.611091798	-1.852435096	-6.897438348	7.94E-09	2.51E-08
Prostate urine vs normal urine	hsamiR1555p	-3.707156301	-1.890312943	-6.473832444	3.70E-08	1.10E-07
Prostate urine vs normal urine	hsalet7g5p	-3.823631097	-1.934943339	-3.796340115	3.92E-04	9.55E-04
Prostate urine vs normal urine	hsamiR1415p	-4.084944166	-2.030316359	-4.712326773	1.93E-05	5.02E-05
Prostate urine vs normal urine	hsamiR2425p	-4.346696563	-2.119919387	-5.174089364	3.90E-06	1.09E-05
Prostate urine vs normal urine	hsamiR2415p	-4.683286247	-2.22752122	-3.359753206	0.001484036	0.003335065
Prostate urine vs normal urine	hsamiR320c	-5.294937761	-2.404613726	-4.187506203	1.12E-04	2.76E-04
Prostate urine vs normal urine	hsamiR10125p	-5.483512722	-2.455100375	-2.60552474	0.011995779	0.023022202
Prostate urine vs normal urine	hsamiR29a5p	-6.018135423	-2.589316571	-3.577487131	7.71E-04	0.00178759
Prostate urine vs normal urine	hsamiR34a5p	-6.494949227	-2.699318248	-4.954144656	8.40E-06	2.22E-05
Prostate urine vs normal urine	hsamiR27b5p	-18.42117906	-4.2032935	-2.523228588	0.014790559	0.028102062
Prostate urine vs normal urine	hsamiR320b	-21.79473629	-4.445907842	-8.740689568	1.04E-11	3.65E-11
Prostate urine vs normal urine	hsamiR165p	-27.19231221	-4.765126926	-4.342464706	6.71E-05	1.70E-04
Prostate urine vs normal urine	hsamiR4235p	-27.82005054	-4.798053136	-2.610022786	0.011857902	0.02298981
Prostate urine vs normal urine	hsamiR30d5p	-32.20291804	-5.009119518	-2.69129083	0.00960358	0.018811136
Prostate urine vs normal urine	hsamiR148a5p	-40.90357873	-5.354155168	-3.124923404	0.002933891	0.00619377
Prostate urine vs normal urine	hsamiR26b5p	-41.56510147	-5.377300828	-3.220619376	0.002229387	0.00481345
Prostate urine vs normal urine	hsamiR320a5p	-60.1225664	-5.909834689	-11.34244734	1.50E-15	4.55E-14
Prostate urine vs normal urine	hsamiR125a5p	-205.7814583	-7.684969186	-5.140296213	4.39E-06	1.19E-05
VHL urine vs normal urine	hsamiR1405p	-2.211942989	-1.145314202	-3.337793073	0.002716283	0.029912925
VHL urine vs normal urine	hsamiR320a5p	-8.617787526	-3.107317529	-5.387475086	1.50E-05	5.72E-04
VHL urine vs normal urine	hsamiR2055p	-10.81397417	-3.43482491	-3.22577821	0.003572432	0.033938109
VHL urine vs normal urine	hsamiR320c	-23.13193143	-4.531813826	-4.480428437	1.52E-04	0.004811174
VHL urine vs normal urine	hsamiR4865p	-29.84470555	-4.899403115	-5.608221802	8.63E-06	4.68E-04
VHL urine vs normal urine	hsamiR320b	-59.06391779	-5.88420515	-8.349093566	1.34E-08	2.55E-06
Cluster 2 urine vs normal urine	hsamiR5745p	-3.601560205	-1.848622021	-3.843511612	0.001354818	0.009533904
Cluster 2 urine vs normal urine	hsamiR148b5p	-3.768213914	-1.913880866	-2.796518525	0.012612798	0.027232177
Cluster 2 urine vs normal urine	hsamiR146b5p	-4.293632278	-2.102198639	-2.749498658	0.013897796	0.029669453
Cluster 2 urine vs normal urine	hsamiR378a5p	-5.262516796	-2.395752933	-4.020627492	9.27E-04	0.007338825
Cluster 2 urine vs normal urine	hsamiR165p	-5.522829311	-2.46540754	-4.594216171	2.74E-04	0.002806596
Cluster 2 urine vs normal urine	hsamiR15p	-7.167293644	-2.841428463	-2.666430949	0.016524348	0.034884734
Cluster 2 urine vs normal urine	hsamiR225p	-7.314545533	-2.87076823	-3.311879726	0.004237125	0.022007556
Cluster 2 urine vs normal urine	hsamiR13p	-7.330505195	-2.873912628	-2.627756042	0.017896048	0.036561819
Cluster 2 urine vs normal urine	hsamiR29b25p	-7.443244235	-2.895931576	-4.048139558	8.74E-04	0.007338825
Cluster 2 urine vs normal urine	hsamiR1455p	-7.555381703	-2.917504643	-5.974249478	1.68E-05	2.65E-04
Cluster 2 urine vs normal urine	hsamiR29b15p	-7.56212133	-2.918790997	-4.03415398	9.01E-04	0.007338825
Cluster 2 urine vs normal urine	hsamiR2245p	-8.058147455	-3.010448205	-4.583438816	2.81E-04	0.002806596
Cluster 2 urine vs normal urine	hsamiR1505p	-8.083081645	-3.01490542	-5.25528988	6.99E-05	8.30E-04
Cluster 2 urine vs normal urine	hsamiR103a15p	-9.059465631	-3.179425956	-2.480689664	0.024170622	0.047344517
Cluster 2 urine vs normal urine	hsamiR103a25p	-9.295974764	-3.216606152	-2.499790842	0.023251209	0.046018017
Cluster 2 urine vs normal urine	hsamiR1405p	-9.508863852	-3.249272974	-2.991065505	0.008382387	0.022007556
Cluster 2 urine vs normal urine	hsalet7d5p	-14.30409977	-3.838356799	-5.277844835	6.68E-05	8.30E-04
Cluster 2 urine vs normal urine	hsamiR320c	-14.68090801	-3.875869296	-3.364469658	0.003786267	0.021158549
Cluster 2 urine vs normal urine	hsamiR2215p	-14.88418317	-3.895708145	-4.18235366	6.56E-04	0.006234635
Cluster 2 urine vs normal urine	hsamiR1555p	-15.65976876	-3.968991004	-6.51348517	6.00E-06	1.04E-04
Cluster 2 urine vs normal urine	hsamiR34a5p	-16.39286826	-4.0349964	-2.927078554	0.009593143	0.02345107
Cluster 2 urine vs normal urine	hsamiR2425p	-20.22897362	-4.338351217	-4.683581074	2.27E-04	0.00254243
Cluster 2 urine vs normal urine	hsamiR1265p	-21.87662851	-4.451318511	-3.13025671	0.006241109	0.022007556
Cluster 2 urine vs normal urine	hsamiR146a5p	-23.97033569	-4.583178208	-5.866223728	2.07E-05	2.81E-04
Cluster 2 urine vs normal urine	hsamiR2055p	-29.53048426	-4.88413311	-2.860988492	0.011021734	0.024070454
Cluster 2 urine vs normal urine	hsamiR199b5p	-31.40544031	-4.972942591	-10.38343361	1.14E-08	7.23E-07
Cluster 2 urine vs normal urine	hsamiR3425p	-32.19979989	-5.008979817	-5.894049539	1.96E-05	2.81E-04
Cluster 2 urine vs normal urine	hsamiR199a5p	-37.72178964	-5.237326219	-11.2268818	3.64E-09	3.46E-07
Cluster 2 urine vs normal urine	hsamiR92a25p	-44.08137474	-5.462097312	-8.362086129	2.39E-07	5.68E-06
Cluster 2 urine vs normal urine	hsamiR23b5p	-48.84544703	-5.610152187	-7.64276941	7.94E-07	1.68E-05
Cluster 2 urine vs normal urine	hsamiR92a15p	-49.3041559	-5.623637353	-8.397036198	2.26E-07	5.68E-06
Cluster 2 urine vs normal urine	hsamiR23a5p	-57.38529039	-5.842609072	-7.05875012	2.21E-06	4.20E-05
Cluster 2 urine vs normal urine	hsamiR4865p	-69.7014474	-6.12311671	-9.283013887	5.65E-08	2.15E-06
Cluster 2 urine vs normal urine	hsamiR320b	-114.2156572	-6.835616625	-9.446243644	4.42E-08	2.10E-06
Cluster 2 urine vs normal urine	hsamiR320a5p	-269.6621418	-8.075009184	-8.600885581	1.63E-07	5.16E-06
Cluster 2 urine vs normal urine	hsamiR1435p	-631.9586624	-9.303686382	-18.63149553	1.50E-12	2.85E-10

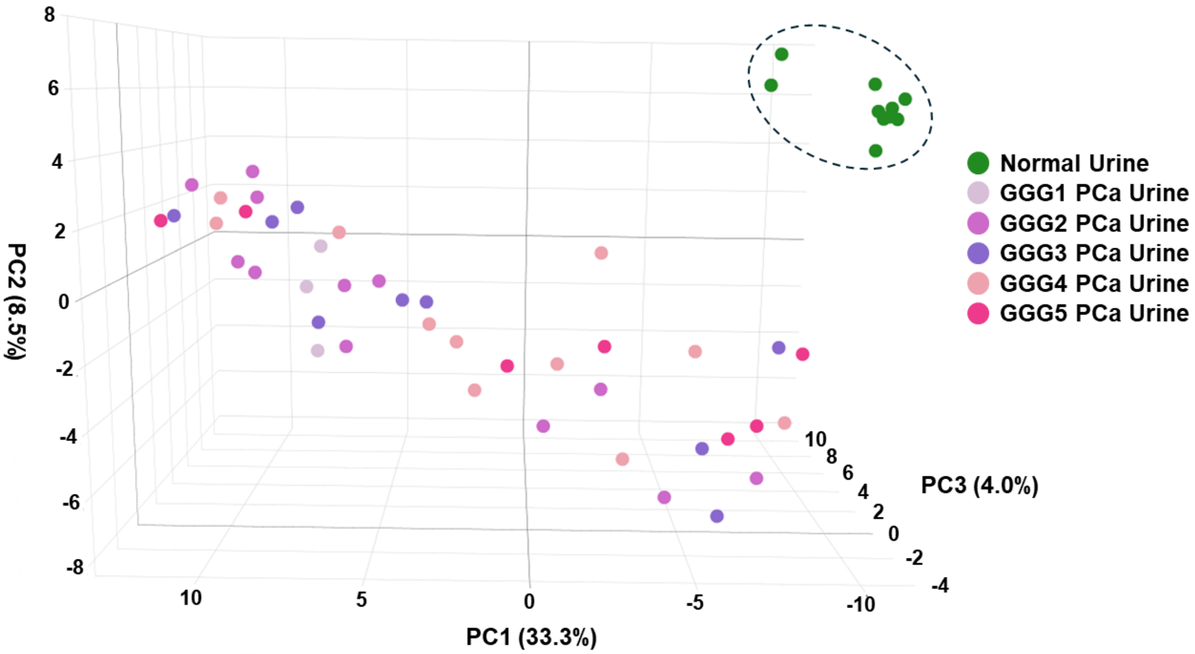

Figure S1: Principal Component Analysis of urine-derived exosomal miRNAs from Prostate cancer patients.

PCA demonstrated distinct differences between the urine-derived exosomal miRNAs present in the normal control individuals (green) and the prostate cancer samples. The urine-derived exosomal miRNA profiles from the prostate cancer patients were color coded to show the Gleason Grade Groups 1-5.

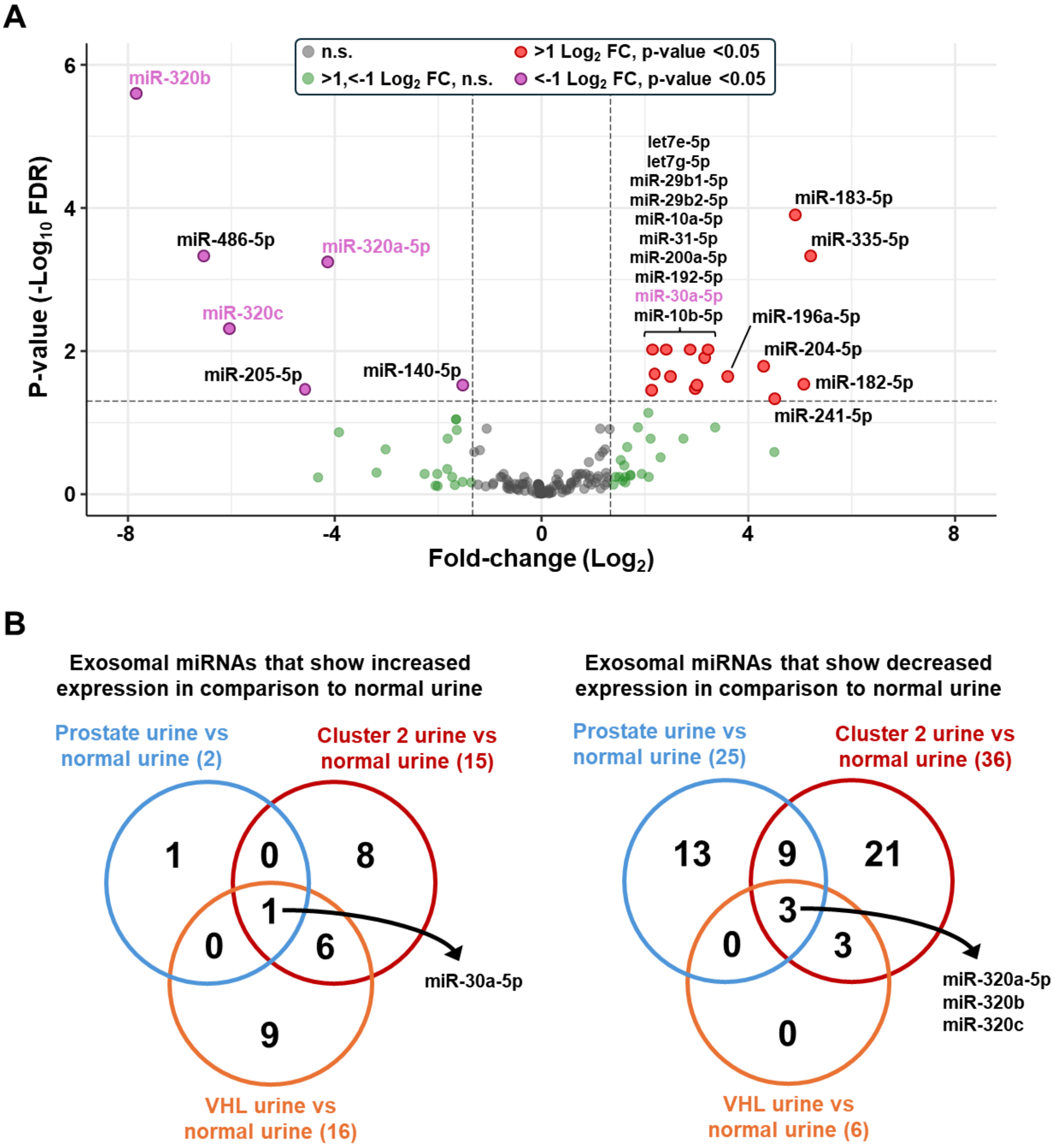
A
n.s.
>1,<-1 Log2 FC, n.s.
>1 Log2 FC, p-value <0.05
<-1 Log2 FC, p-value <0.05
P-value (-Log10 FDR)
Fold-change (Log2)
miR-320b
miR-486-5p
miR-320a-5p
miR-320c
miR-205-5p
miR-140-5p
let7e-5p
let7g-5p
miR-29b1-5p
miR-29b2-5p
miR-10a-5p
miR-31-5p
miR-200a-5p
miR-192-5p
miR-30a-5p
miR-10b-5p
miR-183-5p
miR-335-5p
miR-196a-5p
miR-204-5p
miR-182-5p
miR-241-5p
B
Exosomal miRNAs that show increased expression in comparison to normal urine
Prostate urine vs normal urine (2)
Cluster 2 urine vs normal urine (15)
VHL urine vs normal urine (16)
miR-30a-5p
Exosomal miRNAs that show decreased expression in comparison to normal urine
Prostate urine vs normal urine (25)
Cluster 2 urine vs normal urine (36)
VHL urine vs normal urine (6)
miR-320a-5p
miR-320b
miR-320c

Figure S2: Differential expression analysis of urine-derived exosomal miRNAs from VHL patients with ccRCC and prostate cancer patients.

A – Volcano plot comparing urine-derived exosomal miRNAs from 15 VHL syndrome patients with ccRCC and 10 normal controls. P-values were FDR adjusted and a value of <0.05 was considered significant.

B – Venn diagrams showing the overlaps between the miRNAs that were either upregulated or downregulated in the urine exosomes of all the prostate cancer patient (n=42) or the prostate cancer patients in cluster 2 (n=12) or the VHL syndrome patients with ccRCC (n=15) in comparison to normal control urines (n=10). The miRNAs that were present in all three groups are highlighted.

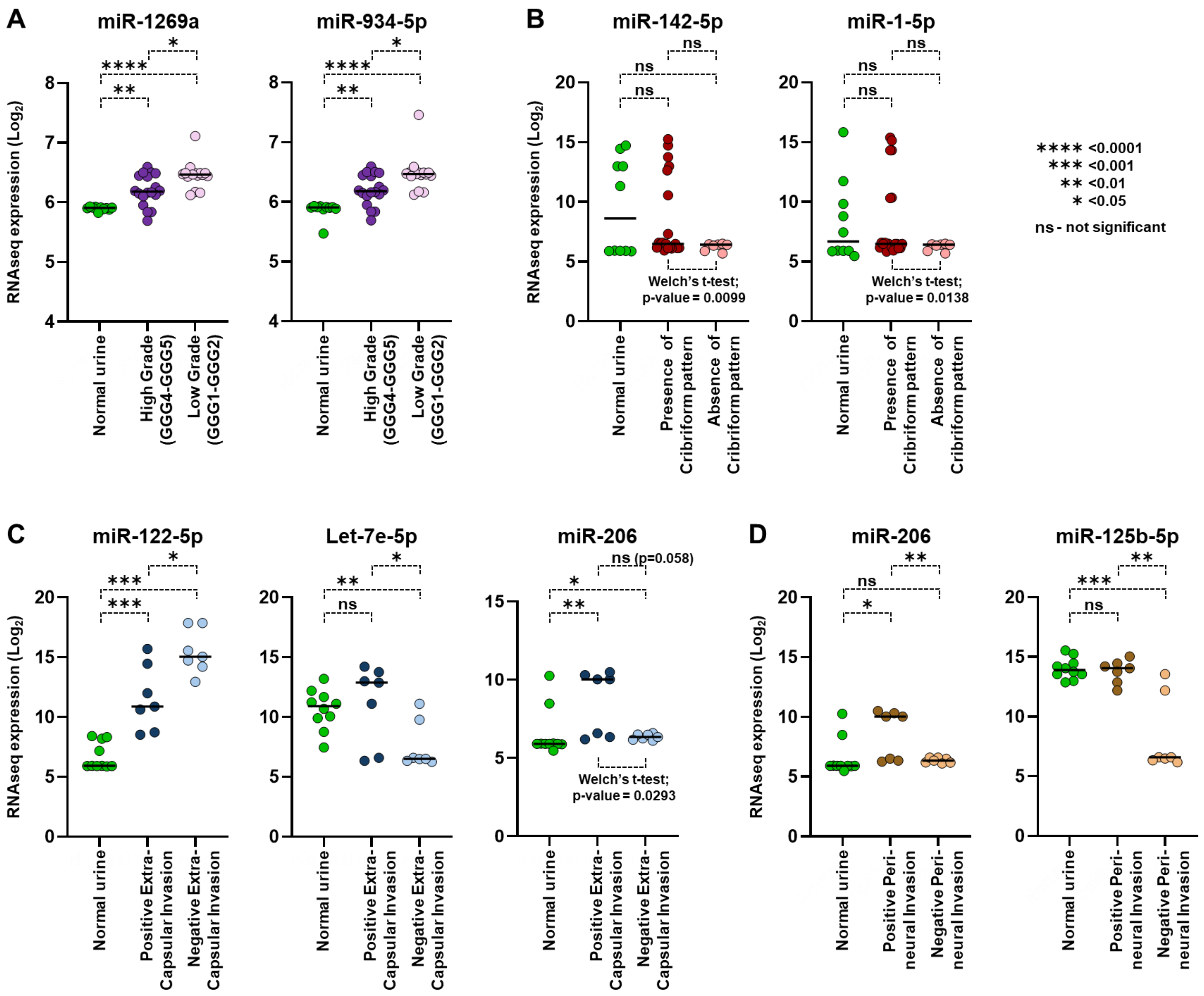

A
miR-1269a
miR-934-5p
RNAseq expression (Log2)
Normal urine
High Grade (GGG4-GGG5)
Low Grade (GGG1-GGG2)
B
miR-142-5p
miR-1-5p
Presence of Cribriform pattern
Absence of Cribriform pattern
Welch's t-test; p-value = 0.0099
Welch's t-test; p-value = 0.0138
**** <0.0001
*** <0.001
** <0.01
* <0.05
ns - not significant
C
miR-122-5p
Let-7e-5p
miR-206
Positive Extra-Capsular Invasion
Negative Extra-Capsular Invasion
ns (p=0.058)
Welch's t-test; p-value = 0.0293
D
miR-206
miR-125b-5p
Positive Peri-neural Invasion
Negative Peri-neural Invasion

Figure S3: Additional differential expression analysis associated with clinical features in prostate cancer patients.

A – Differentially expressed miRNAs associated with high grade tumors (GGG4-5).

B – Differentially expressed miRNAs associated with high grade tumors (GGG4-5).

C & D – Differentially expressed miRNAs associated with either extracapsular or perineural invasion.

∗∗∗∗ - <0.0001, ∗∗∗ - <0.001, ∗∗ - <0.01, ∗ - <0.05, ns – not significant.
